# Utility of Tetrahydrobiopterin Pathway in the Assessment of Diabetic Foot Ulcer: Significant and Complex Interrelations

**DOI:** 10.1155/2019/3426878

**Published:** 2019-11-16

**Authors:** Marwan Al-Nimer, Rawa Ratha, Taha Mahwi

**Affiliations:** ^1^Department of Pharmacology and Toxicology, Hawler Medical University, Erbil, Iraq; ^2^Department of Clinical Pharmacy, University of Sulaimani, Sulaimani, Iraq; ^3^Department of Pharmacology and Toxicology, College of Pharmacy, University of Sulaimani, Sulaimani, Iraq; ^4^Department of Medicine, College of Medicine, University of Sulaimani, Sulaimani, Iraq

## Abstract

**Objectives:**

Tetrahydrobiopterin (BH4) pathway that included generation of neopterin (Neop), biopterin (Biop), and nitric oxide (NO) is altered in type 2 diabetes (T2D). The aim of this study was to assess the biomarkers of BH4 pathway in noninfected DFUs and to relate these levels to the variables of diabetes as well as to the hematological indices.

**Methods:**

We performed a cross-sectional investigating study in a Kurdish people including 30 healthy subjects (group I), 66 T2D patients (group II), and 57 DFUs patients (group III). Hematological indices including red cell distribution width (RDW), mean platelet volume (MPV), and platelet distribution width (PDW) were determined by Coulter hematological analysis. Serum BH4 markers including NO, Neop, and Biop were determined by using an enzyme-linked immunosorbent assay (ELISA) technology. The relationship between BH4 markers with glycemic and hematological indices was assessed by Spearman's correlation and multivariable regression analysis.

**Results:**

Neop was significantly increased while PDW was significantly decreased in group III compared with group II patients. Nitric oxide was found to be inversely correlated with age (*r* = −0.382), duration of diabetes (*r* = −0.264), mean arterial blood pressure (*r* = −0.532), body mass index (*r* = −0.321), RDW (*r* = −0.322), and PDW (*r* = −0.284) in group III patients. Circulating Neop and Biop significantly correlated with RDW and erythrocyte sedimentation rate. Multivariable regression analysis revealed that serum Neop predicted the DFUs in 92.5% of group III patients.

**Conclusion:**

Tetrahydrobiopterin biomarkers are predictors of DFUs and the significant correlation of neopterin with red distribution width and erythrocyte sedimentation rate indicating the role of neopterin in the vascular and inflammation concerns of noninfected DFUs.

## 1. Introduction

Diabetic foot syndrome (DFS) is one of the serious complications of diabetes mellitus that adversely affects the quality of life [1]. One meta-analysis study mentioned that the prevalence of diabetic foot ulcers (DFUs) is 6.3%, and it is more common in men with type 2 diabetes (T2D) [2]. Multifactorial risk factors and comorbidities including neuropathy, abnormal vascular response, metabolic derangement, trauma, and infections are involved in the development of DFS [3–5]. There is evidence that platelet indices, including mean platelet volume (MPV), and mean platelet distribution width (PDW), were significantly low in septic DFUs indicating their role in the pathogenesis of DFUs [6]. Other authors suggested that MPV, which increases in T2D, is a risk factor for peripheral artery disease that is associated with T2D [7]. Red distribution width (RDW) percentage was significantly increased in complicated diabetic patients and directly correlated with glycated hemoglobin (HbA1c) [8]. There is an inverse relationship between the level of nitric oxide (NO) with the MPV and RDW, as low levels of nitrites were associated with aggregation of red cells and platelets, which may be prone to the development of peripheral artery disease [9, 10]. Tetrahydrobiopterin (BH4) pathway that included generation of neopterin (Neop), biopterin (Biop), and nitric oxide (NO) is involved in many pathological conditions including T2D [11]. The previous study reported the mean ± SD of the serum level of NO in patients with diabetic foot ulcer was 17.6 ± 7.6 *μ*mol which is higher than the corresponding value of diabetic patients without foot ulcers (11.8 ± 7.8 *μ*mol) or healthy subjects (6.4 ± 2.0 *μ*mol) [12]. Other researchers suggested that a low level of NO, due to deficiency of NO-synthase, caused ischemia of the peripheral nerves which led to peripheral neuropathy and thereby DFUs [13]. Cumulative evidence showed that serum level of Neop served as a biomarker of T2D with or without DFUs [14, 15]. Our hypothesis is that disturbances of BH4 pathway in diabetic patients may induce several changes at the vascular, neuronal, and immunological leads or associates with DFUs.

In the last years, authors reported the role of the inflammation and the endothelial dysfunction in DFUs manifested by a significant production of Neop and reducing the synthesis of NO [11, 16]. In this study, the authors addressed the role of tetrahydrobiopterin pathway which included Neop, Biop, and NO as contributors to the endothelial dysfunction and inflammation in DFUs.

Therefore, the aim of this study is to assess the serum levels of the BH4 pathway biomarkers, including neopterin, biopterin, and nitric oxide, in diabetic patients with and without noninfected DFUs and to relate these levels to the variables of diabetes as well as to the hematological indices.

## 2. Materials and Methods

### 2.1. Ethical Approval

The present randomized cross-sectional study was conducted in Shar Teaching Hospital in cooperation with the College of Medicine at the University of Sulaimani in Sulaimani city, Iraq through 2018. The Ethical Committee of the University of Sulaimani approved this study with a registration number 7.29, 3275 in 12-12-2018. All procedures were conducted in accordance with the Declaration of Helsinki, and patients were informed that they were free to withdraw from the study at any time. The researchers explained the study design and obtained the informed consent from each participant for the laboratory investigations, before the study began.

### 2.2. Participants

This study was conducted on patients with T2D who were referred to the Consultant Clinics at Shar Teaching hospital in Sulaimani city, Iraq. Eligible patients were both genders aged > 35 years old. The patients were allocated randomly by using randomized tables. Patients with noninfected DFUs (proved by laboratory culture and sensitivity testing) according to the Wagner-Meggitt classification (grade 0-2) and patients without DFUs were included in the study [17]. Criteria of exclusion were patients with current ischemic heart disease, complicated diabetes (retinopathy, deterioration of renal function), pregnancy, associated blood disorders, and terminal illness.

### 2.3. Sample Size

A pilot study was done to estimate the sample size. The mean, standard deviation, and the difference between the means were calculated from the pilot study. The power of the study 1 − *β* is fixed at 80% (0.8) and the significance level is fixed at 5% (≤0.05). Then, the following equations were used to calculate the sample size:

(1) $$\begin{array}{c} \text{sample size per group}=1+2C\times {\left(\text{standard deviation}/\text{difference between means}\right)}^{2}, \end{array}$$

where *C* represents the constant value which equals to 7.85 when the 1 − *β* = 0.8 and *α* = 0.05.

### 2.4. Study Measurements

The disease-related information was ascertained from subjective responses to the questionnaire administered by the authors. Body mass index (BMI) was calculated by using Quetelet's equation that equals to the body weight (kg) by squared the height (m). Blood pressure (BP) was measured as a mean of three readings at rest over 5 minutes. Participants who were smokers were excluded from the study. Mean arterial BP was calculated by using the following equation:

(2) $$\begin{array}{c} \text{mean arterial BP}\left(\text{mmHg}\right)=\text{diastolic BP}+\left(\frac{1}{3}\times \left[\text{systolic BP minus diastolic BP}\right]\right). \end{array}$$

A total number of 153 participants were included in the study: 30 healthy subjects (served as a negative control; group I), 66 patients without DFUs served as a reference group (group II), and 57 patients with DFUs (group III).

After an overnight fasting, venous blood was drawn from each participant to determine the hematological and biochemical parameters. Venous blood was divided into two portions: the first portion was kept in anticoagulant test tubes for the determination of glycated hemoglobin (HbA1c) and hematological indices, including red cell distribution width (RDW), mean platelet volume (MPV), platelet distribution width (PDW), and erythrocyte sedimentation rate (ESR). Hematological indices were measured by using Coulter hematological analysis, and the percentage of HbA1c was determined by using the HbA1c assay kit. The second portion was kept in plain test tubes, centrifuged at 3,000 rpm for 15 minutes, the sera separated for determination of serum BH4 markers including NO, Neop, and Biop by using an enzyme-linked immunosorbent assay (ELISA) technology according to the instruction of the manufacturer.

### 2.5. Statistical Analysis

Data for continuous variables are presented as mean ± SD, and the categorical variables are presented as number and percentage. Statistical comparisons between continuous parameters were performed by one-way analysis of variance (ANOVA) with the *post hoc Bonferroni* test and independent two-sample *t* test. Statistical comparisons between categorical variables were performed using chi-squared test. Relationships between continuous variables were performed by using Spearman's correlation, and the predictions of the dependent variable were calculated by using multivariable regression analysis with ANOVA. The prediction equation was derived from the constant (*a*) and the summation of the *β* coefficient multiplying with the value of each independent variable; *P* values were two-tailed and considered significant when ≤0.05.

## 3. Results

Table 1 shows the characteristics of the patients. There is an insignificant difference in sex distribution or the family history of diabetes between group II and group III.

**Figure 1:**
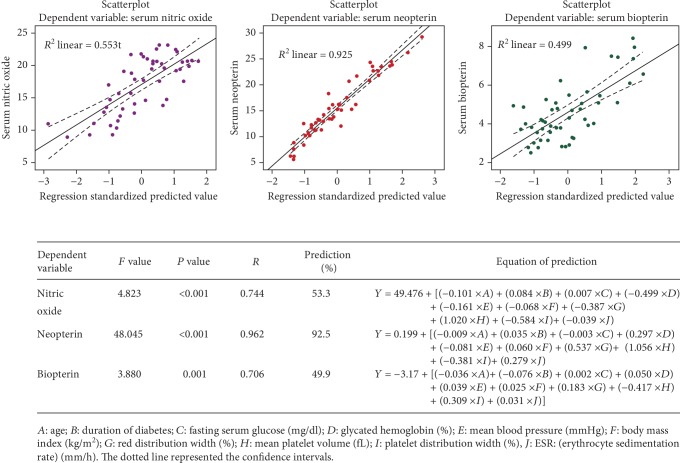
Prediction of the determinants of tetrahydrobiopterin pathway in diabetic foot ulcer.

The means ± SDs of the age and the duration of diabetes were significantly higher in Group III than the corresponding values of the group I and group II. Group III patients have a significant long duration of diabetes, and they are older than group II patients. Previous history of amputation was observed in 15.78% of group III patients, and the current presentation of diabetic ulcers showed that distribution of patients with grades 0, 1, and 2 were 21.1%, 50.9%, and 28%, respectively, of group III patients (Table 1). Diabetic patients (groups II and III) have a significant high systolic BP compared with group I, and the diastolic BP of group II was significantly higher than the corresponding level of group III patients (Table 1). There is an insignificant difference between groups II and III in the fasting serum glucose level and HbA1c %. Diabetic patients of groups II and III have a significant high RDW percentage than healthy subjects (group I), and there is a nonsignificant difference in the PDW and MPV between healthy subjects and diabetic patients (Table 2). Group III patients have a significantly lower percentage of PDW than the corresponding percentage of the group II patients (Table 2). The mean level of ESR of group III was significantly higher than the corresponding levels of groups I and II. Assessment of the BH4 pathway markers showed significant low serum levels of NO and Biop among group II and III patients compared with group I while the serum levels of Neop were significantly higher in group II and III patients (Table 3). The mean serum level of Neop among group III patients is significantly increased by 4.31 nmol/L than the mean serum level of Neop in group II patients (Table 3). The ratio of Neop-to-Biop is significantly higher among group III patients compared with those of group I and II.  Table 4 shows the interrelationship between the markers of BH4 pathway with the different variables assessed in this study. Serum nitric oxide is significantly correlated with many factors in group III while mean arterial BP in group II. Significant positive correlations between serum Neop with PDW and ESR were observed in group II patients while in group III, the positive correlation between serum Neop with RDW and ESR were observed (Table 4). A serum Biop level significantly increased as the ESR or the serum Neop level increased in both group II and III patients (Table 4). Moreover, the serum Biop is inversely related to the serum nitric oxide and directly to RDW among group III patients. Multivariable regression analysis revealed that serum level of Neop is a good predictor of multivariables that are related to DFUs, which accounted for 92.5%, while prediction percentages of serum nitric oxide and Biop were 53.3% and 49.9%, respectively (Figure 1). The biomarkers of the BH4 pathway were significantly correlated with several factors that related to diabetic ulcer, and Figure 1 shows the predicted equations.

## 4. Discussion

The present results showed significant abnormalities in the PDW, and RDW values in the DFU patients compared with healthy subjects and patients without DFUs. Significant higher serum level of Neop and lower serum levels of Biop and NO were observed in Group III. Serum Neop is a significant predictor of the DFUs taking consideration the multifactors that participated in the development of DFUs. This study showed that longer duration of diabetes and older age of patients are commonly associated with DFUs [4]. Patients with DFUs were significantly overweight, have nonsignificantly lower mean arterial BP, and have high fasting serum glucose. Our findings are in agreement with Khan et al.'s study which found that greater BMI is significantly associated with foot ulcer while high BP or T2D is insignificantly linked to FUs [18]. Significantly high red cell distribution width value in DFUs is in agreement with other studies that demonstrated that high RDW is a marker of complicated T2D [8, 19]. A low value of PDW and high ESR level are significant discriminators between noninfected DFUs and non-DFU patients while MPV values in both groups II and III do not show a significant difference. A recent study demonstrated that MPV and PDW are diagnostic markers of diabetic complications as they insignificantly increased compared with uncomplicated diabetes [20]. This study demonstrates that there is a reciprocal change between RDW and PDW in noninfected DFUs. High serum levels of Neop in DFS were reported previously by Al-Nimer and Dezayee (2011), suggesting its role in the activation of the immune system, and this explained the significant correlation between neopterin with the ESR in this study [15]. Previous study demonstrated a significant high serum Neop level among T2D compared with healthy subjects [14] while this study demonstrated that the mean serum level of Neop in DFUs is significantly higher than that of non-DFU patients and it correlates significantly and directly with the RDW and ESR. It is expected to find a significant high value of Neop in group III because DFUs are associated with inflammation and activation of oxidative stress [21, 22]. Moreover, recent studies find that serum level of Neop is a useful predictor of diabetic neuropathy in pediatric T1D [23]. Low serum levels of Biop and high serum Neop-to-Biop ratio in diabetes are expected as Biop is a physiological endogenous antagonist of Neop. Low serum NO in DFUs that is demonstrated in this study agreed with previous studies that low serum NO contributed to the pathogenesis of peripheral vascular disease that is associated with DFUs [24, 25].  Table 4 shows the complex interrelations between the BH4 markers and other indices indicating that BH4 pathway subjected to significant disturbances in DFUs which can be used as predictors in the DFS as illustrated in Figure 1. Disturbances of the BH4 pathway are reflected by an increase of Neop levels and a decrease levels of the Biop and NO. Therefore, both the inflammatory process and nitrative stress are shared concomitantly in the pathogenesis of DFUs [26, 27]. Oral antidiabetic agents, as well as short-term therapy of insulin that are prescribed to the patients, are not influencing the results because the serum levels of glucose and glycated hemoglobin indicated that the patients were poorly controlled.

The strength of this study is providing a significant predictive equation for BH4 markers which can be applied at 92% with Neop, 53.3% with NO, and 49.9% with Biop in DFU patients. One of the limitations of the study is that it was not registered in a public clinical trial because the policy of research work in our university is to register the proposal at the Scientific Committee of the University; otherwise, the university does not consider this research as an activity of the researcher.

We conclude that BH4 biomarkers are valuable predictors of DFUs and their associated factors. Neopterin is significantly correlated with RDW and ESR indicating the role of neopterin in the vascular and inflammation concerns of the noninfected DFUs.

## Figures and Tables

**Table 1:** Characteristics of the participants.

Determinants	Group I (*n* = 30)	Group II (*n* = 66)	Group III (*n* = 57)	ANOVA test		Post hoc test; comparison between groups
				*F* value	*P* value	*p* value
Sex (male : female)	10 : 20	32 : 34	16 : 41			0.056
Age (year)	50.1 ± 6.4	54.0 ± 8.2	56.7 ± 8	7.243	0.001	0.067^∗^; 0.001^†^; 0.169^‡^
Duration of diabetes	—	7.8 ± 4.3	10.31 ± 5.86	7.155	0.009	0.009^‡^
Family history of diabetes	0	43	36			0.818^‡^
Previous history of amputation	0	0	9			0.001^‡^
Body mass index	29.4 ± 3.0	28.4 ± 4.1	39.0 ± 5.8	87.358	<0.001	0.973^∗^; <0.001^†^; <0.001^‡^
Wagner-Meggitt grades						
Grade 0	0	0	12			<0.001^‡^
Grade 1	0	0	29			
Grade 2	0	0	16			
Blood pressure (mmHg)						
Systolic	112.3 ± 7.3	131.1 ± 20.0	131.2 ± 20.1	12.680	<0.001	<0.001^∗^; <0.001^†^; 1.000^‡^
Diastolic	75.2 ± 5.0	81.0 ± 9.8	76.5 ± 10.3	5.495	0.005	<0.015^∗^; 1.000^†^; 0.027^‡^
Mean	87.5 ± 5.2	97.7 ± 12.2	93.1 ± 17.4	8.397	<0.001	0.001^∗^; 0.016^†^; 0.451^‡^
Fasting serum glucose (mg/dl)	86.9 ± 8.4	195.1 ± 65.7	220.9 ± 80.6	42.772	<0.001	<0.001^∗^; <0.001^†^; 0.094^‡^
Glycated hemoglobin (%)	4.66 ± 0.29	9.32 ± 2.10	9.38 ± 1.73	90.891	<0.001	<0.001^∗^; <0.001^†^; 1.000^‡^

The results are expressed as mean ± SD. ^∗^Comparison between groups I and II; ^†^comparison between group I and III; ^‡^comparison between group II and III *P* value was calculated by ANOVA test, *post hoc Bonferroni* test, independent two-sample *t* test (for duration of diabetes) for continuous data, and chi-square test for categorized data (sex variable). Group I: healthy subjects; group II: nondiabetic ulcer patients; group IIL: diabetic ulcer patients.

**Table 2:** Hematological indices.

Determinants	Group I (*n* = 30)	Group II (*n* = 66)	Group III (*n* = 57)	ANOVA test		*Post hoc* test; comparison between groups
				*F* value	*P* value	*p* value
Red cell width distribution	11.81 ± 0.81	12.52 ± 1.334	12.66 ± 1.28	4.971	0.008	0.030^∗^; 0.008^†^; 1.000^‡^
Platelet width distribution	12.59 ± 1.20	13.32 ± 1.85	12.55 ± 1.62	3.907	0.022	0.143^∗^; 1.000^†^; 0.034‡
Mean platelet volume	8.54 ± 0.86	8.6 ± 1.5	8.45 ± 1.01	0.246	0.783	1.000^∗^; 1.000^†^; 1.000^‡^
Erythrocyte sedimentation rate (mm/h)	11.9 ± 5.5	14.01 ± 11.71	29.0 ± 19.38	21.440	<0.001	1.000^∗^; <0.001^†^; <0.001^‡^

The results are expressed as mean ± SD. ^∗^Comparison between groups I and II; ^†^comparison between groups I and III; ^‡^comparison between groups II and III. *P* value was calculated by ANOVA test, *post hoc Bonferroni* test, for continuous data. Group I: healthy subjects; group II: nondiabetic ulcer patients; and group III: diabetic ulcer patients.

**Table 3:** Biochemical analysis of tetrahydrobiopterin pathway.

Determinants	Group I (*n* = 30)	Group II (*n* = 66)	Group III (*n* = 57)	ANOVA test		*Post hoc* test; comparison between groups
				*F* value	*P* value	*p* value
Serum nitric oxide (*μ*mol/L)	25.11 ± 5.22	18.91 ± 4.40	17.30 ± 4.15	30.346	<0.001	<0.001^∗^; <0.001^†^; 0.149^‡^
Serum neopterin (nmol/L)	7.49 ± 2.17	11.60 ± 5.06	15.91 ± 5.71	52.305	<0.001	0.001^∗^; <0.001^†^; <0.001^‡^
Serum biopterin (nmol/L)	8.12 ± 1.13	5.09 ± 1.55	4.85 ± 1.64	70.874	<0.001	<0.001^∗^; <0.001^†^; 1.000^‡^
Ratio of neopterin/biopterin	0.937 ± 0.302	2.33 ± 0.79	3.45 ± 1.27	7.155	0.009	<0.001^∗^; <0.001^†^; <0.001^‡^

The results are expressed as mean ± SD. ^∗^Comparison between groups I and II; ^†^comparison between groups I and III; ^‡^comparison between groups II and III. *P* value was calculated by ANOVA test, *post hoc Bonferroni* test, for continuous data. Group I: healthy subjects; group II: nondiabetic ulcer patients; and Group III: diabetic ulcer patients.

**Table 4:** Correlations between the determinants of tetrahydrobiopterin pathway and variables related to the diabetes in patients with diabetes foot ulcer.

Determinants	Group II			Group III		
	Nitric oxide (*μ*mol/L)	Neopterin (nmol/L)	Biopterin (nmol/L)	Nitric oxide (*μ*mol/L)	Neopterin (nmol/L)	Biopterin (nmol/L)
Age (year)	-0.235	0.004	0.103	-0.382	0.121	0.1033
	0.067	0.974	0.409	0.004	0.369	0.409

Duration (year)	-0.099	0.104	0.215	-0.264	-0.041	0.227
	0.428	0.406	0.084	0.050	0.760	0.090

Fasting serum glucose (mg/dl)	-0.126	-0.162	0.085	0.062	0.008	0.064
	0.312	0.193	0.499	0.651	0.952	0.636

HbA1c%	-0.171	0.126	0.193	-0.243	-0.030	0.240
	0.176	0.322	0.126	0.089	0.834	0.089

Mean arterial blood pressure (mmHg)	-0.246	-0.014	0.103	-0.532	-0.089	0.212
	0.046	0.914	0.408	<0.001	0.516	0.116

Body mass index (kg/m^2^)	0.100	0.122	0.095	-0.321	0.130	0.021
	0.427	0.331	0.449	0.016	0.336	0.877

Red cell distribution width CV (%)	0.026	0.142	0.001	-0.322	0.310	0.306
	0.837	0.254	0.995	0.012	0.019	0.021

Mean platelet volume (fL)	-0.009	-0.085	-0.169	-0.179	0.091	-0.060
	0.940	0.498	0.175	0.187	0.501	0.658

Platelet distribution width (%)	0.048	0.333	0.198	-0.284	0.204	0.047
	0.700	0.006	0.111	0.034	0.128	0.728

Erythrocyte sedimentation rate (mm/h)	-0.134	0.897	0.539	-0.191	0.933	0.505
	0.282	<0.001	<0.001	0.160	<0.001	<0.001

Serum nitric oxide (*μ*mol/L)		-0.055	-0.154		-0.129	-0.321
		0.660	0.218		0.343	0.016

Serum neopterin (nmol/L)			0.518			0.540
			<0.001			<0.001

The results are expressed as Spearman correlation factor (above) and *p* value (below) in each cell of the table.

## Data Availability

The data used to support the findings of this study are available from the corresponding author upon request.
